# Supplementary material for: Incidence, Risk Factors, and Outcomes of Symptomatic Bone Cement Displacement following Percutaneous Kyphoplasty for Osteoporotic Vertebral Compression Fracture: A Single Center Study
Source: J Clin Med. 2022 Dec 19;11(24):7530. doi: 10.3390/jcm11247530 (PMC9783083; doi:10.3390/jcm11247530)
Supplement: Supplementary file 1 [file jcm-11-07530-s001.zip › jcm-2025001-supplementary.pdf]

STROBE statement—a checklist of items that should be included in reports of observational studies.

	Item No.	Recommendation	Page No.	Relevant text from manuscript
**Title and abstract**	1	(*a*) Indicate the study's design with a commonly used term in the title or the abstract	1	Line 23 "retrospective study"
		(*b*) Provide in the abstract an informative and balanced summary of what was done and what was found	1–2	Lines 23–49
		**Introduction**		
Background/rationale	2	Explain the scientific background and rationale for the investigation being reported	2	Lines 54–76
Objectives	3	State the specific objectives, including any prespecified hypotheses	2	Lines 76–83
		**Methods**		
Study design	4	Present the key elements of the study's design early in the paper	2–3	Lines 86–94 and Figure 1
Setting	5	Describe the setting, locations, and relevant dates, including periods of recruitment, exposure, follow-up, and data collection	2–3	Lines 86–94 and Figure 1
Participants	6	(*a*) *Cohort study*: Give the eligibility criteria, the sources and methods of participant selection, and describe methods of follow-up<br>*Case-control study*: Give the eligibility criteria, the sources and methods of case ascertainment and control selection, and the rationale for the choice of cases and controls<br>*Cross-sectional study*: Give the eligibility criteria and the sources and methods of participant selection	3	Figure 1
		(*b*) *Cohort study*: For matched studies, give matching criteria and the number of exposed and unexposed<br>*Case-control study:* For matched studies, give matching criteria and the number of controls per case		
Variables	7	Clearly define all outcomes, exposures, predictors, potential confounders, and effect modifiers, and give diagnostic criteria if applicable	2–3	Lines 94–101 and Figures 1 and 2
Data sources/ measurement	8*	For each variable of interest, give sources of data and details of methods of assessment (measurement), and describe the comparability of assessment methods if there is more than one group	3–6	Lines 114–170, Figures 2, 3, 4, and 5
Bias	9	Describe any efforts to address potential sources of bias	7	Lines 194–197
Study size	10	Explain how the study size was arrived at	3	Figure 1

Continued on the next page

**Quantitative variables**	**11**	**Explain how quantitative variables were handled in the analyses, and if applicable, describe which groupings were chosen and why**	**7**	**Lines 186–194**
Statistical methods	12	(*a*) Describe all statistical methods, including those used to control for confounding	7	Lines 184–198
		(*b*) Describe any methods used to examine subgroups and interactions	7	Lines 184–198
		(*c*) Explain how missing data were addressed	3	Figure 1
		(*d*) *Cohort study:* If applicable, explain how the loss to follow-up was addressed *Case-control study*: If applicable, explain how the matching of cases and controls was addressed *Cross-sectional study:* If applicable, describe analytical methods taking the sampling strategy into account	3	Figure 1
		(*e*) Describe any sensitivity analyses		
		**Results**		
Participants	13*	(a) Report the number of individuals at each stage of the study (e.g., numbers potentially eligible, examined for eligibility, confirmed eligible, included in the study, completing follow-up, and analyzed)	3	Figure 1
		(b) Give reasons for non-participation at each stage	3	Figure 1
		(c) Consider the use of a flow diagram	3	Figure 1
Descriptive data	14*	(a) Give characteristics of study participants (e.g., demographic, clinical, social) and information on exposures and potential confounders	7	Lines 200–209, Table 2, and Figure 6
		(b) Indicate the number of participants with missing data for each variable of interest	3	Figure 1
		(c) *Cohort study:* Summarize follow-up time (e.g., average and total amount)	3	Figure 1
Outcome data	15*	*Cohort study*: Report numbers of outcome events or summary measures over time	*9*	Lines 236–243 and Table 3
		Case-control study: Report numbers in each exposure category or summary measures of exposure		
		Cross-sectional study: Report numbers of outcome events or summary measures		
Main results	16	(*a*) Give the unadjusted estimates and, if applicable, confounder-adjusted estimates and their precision (e.g., 95% confidence interval); make clear which confounders were adjusted for and why they were included	10	Lines 247–256 and Table 4
		(*b*) Report category boundaries when continuous variables were categorized	8–9	Lines 216–243 and Tables 2 and 3
		(*c*) If relevant, consider translating estimates of relative risk into absolute risk for a meaningful time period		

Continued on the next page

Other analyses	17	**Report other analyses done (e.g., analyses of subgroups and interactions and sensitivity analyses)**	**7–8 and 10**	**Lines 207–209, 260–269, and Tables 1 and 5**
		**Discussion**		
Key results	18	Summarize key results with reference to study objectives	11	Lines 278–291
Limitations	19	Discuss the limitations of the study, taking into account sources of potential bias or imprecision and both the direction and magnitude of any potential bias	14	Lines 428–433
Interpretation	20	Give a cautious overall interpretation of results considering objectives, limitations, the multiplicity of analyses, results from similar studies, and other relevant evidence	12–14	Lines 306–427
Generalizability	21	Discuss the generalizability (external validity) of the study results	12–14	Lines 306–427
**Other information**				
Funding	22	Give the source of funding and the role of the funders for the present study and for the original study on which the present article is based, if applicable	15	Lines 448–449

*Give information separately for cases and controls in case-control studies and, if applicable, for exposed and unexposed groups in the cohort and cross-sectional studies.

Note: An explanation and elaboration article discusses each checklist item and gives methodological background and published examples of transparent reporting. The STROBE checklist is best used in conjunction with this article (freely available on the Web sites of PLoS Medicine at http://www.plosmedicine.org/, Annals of Internal Medicine at http://www.annals.org/, and Epidemiology at http://www.epidem.com/). Information on the STROBE Initiative is available at www.strobe-statement.org.
